# Assessment of Immune Status in Dynamics for Patients with Cancer Undergoing Immunotherapy

**DOI:** 10.1155/2021/6698969

**Published:** 2021-05-03

**Authors:** Bacinschi Xenia Elena, Laurentia Nicoleta Gales, Anca Florina Zgura, Laura Iliescu, Rodica Maricela Anghel, Bogdan Haineala

**Affiliations:** “Carol Davila” University of Medicine and Pharmacy, 8 Eroii Sanitari Bvd., Bucharest 050474, Romania

## Abstract

Immunotherapy using immune checkpoint inhibitors has revolutionized the treatment, and many types of cancer show a response rate of 20–40% and a significant increase in five-year survival. However, immunotherapy is expensive and may cause serious adverse events. Therefore, a predictive method allowing identification of responding patients before starting the treatment would be very useful. In this study, we aimed to identify and implement other individual prognosis factors, factors that could lead to an improved clinical decision made in regard to the patient to establish an individualized treatment. *Materials and Methods*. All patients recruited from October 2018 to July 2019 were treated in OncoFort Hospital, Bucharest, with nivolumab or pembrolizumab. We investigated T lymphocyte CD3+, CD4+, CD8+, and CD4/CD8 cells by flow cytometry in patients before and after receiving treatment with anti-PD-1 agents. *Results*. We found that the responder group showed higher expression on CD4+ cells than the nonresponder group after the first cycle of immunotherapy. The prediction of the immunotherapeutic effect revealed that the elevation of T lymphocytes CD8+ and CD4+ after the first cycle of immunotherapy was followed by a decrease in their expression after the second cycle and was followed by a return almost to that one after the first administration. *Conclusion*. Our work indicates that the evaluation of the cells of the immune system in relation to the tumor and immunotherapy may lead to a better understanding of the pathogenic mechanisms and the identification of prognostic and predictive factors that will more effectively model the therapeutic approach.

## 1. Introduction

Cancer has been the second leading cause of mortality after cardiovascular disease for several years, and, in some countries, it has become the leading cause of death recently. Immunotherapy, using immune checkpoint inhibitors, has revolutionized the treatment, and many types of cancer show a response rate of 20–40% and a significant increase in five-year survival. However, immunotherapy is expensive and may cause serious adverse events [1, 2]. Therefore, a predictive method allowing identification of responding patients before starting the treatment would be very useful. There is also an urgent need for a method to predict cancer recurrence after surgery, which might be used to provide patients at risk with adjuvant immuno- or chemotherapy [3].

Over 83,000 new cases of cancer were recorded in Romania in 2018, and 50,900 Romanians died because of this disease in 2017, according to data from the Global Cancer Observatory [3].

Lung cancer continues to have the highest incidence in Romania, namely, 13.6% of new cases, followed by colon cancer (13.3%) and breast cancer (11.5%) [5]. According to the latest WHO data published in 2017, lung cancer deaths in Romania reached 10,458 or 4.42% of total deaths. According to the latest WHO data, published in 2017, skin cancer deaths in Romania reached 1,000 or 0.42% of total deaths. The death rate is 2.91 per 100,000 of population ranks in Romania and 29 in the world [6]. In Romania, late diagnosis is prevalent in several cancer types. In lung cancer, for instance, 7 from 10 Romanian patients, such a disease is found out only in its 4th stage. Thus, small-cell lung cancer, renal cancer, and melanoma represent a major health challenge in Romania, with an enormous socioeconomic impact. In recent years, the treatment of many types of cancer, including NSCLC, melanoma, and renal cancer, has been revolutionized with the advent of immune checkpoints. Immune checkpoints are receptors that are involved in modulating the activation of immune cells to limit the duration and intensity of the reaction immune. It exists on the surface of one coactivating receptor cell (which strengthen activation) and receptor coinhibitors (which decrease activation). This is a complex balance between activating signals and inhibitory signals that determine whether an immune cell can activate [7]. Thus, when one T lymphocyte recognizes its specific antigen through its antigenic receptor (TCR), it can only be activated if the different signals sent by its point's controls are in favor of activation. Thus when one T lymphocyte recognizes its specific antigen through its antigenic receptor (TCR), it can only be activated if the different signals sent by its point's controls are in favour of activation. This phenomenon is important from physiological point. He intervenes to preventing the risk of autoimmunity (inhibitory receptors) but also strengthens activation of the immune system. It also prevents the excessive reaction of the immune system: when an immune response occurs, the inflammatory signals released into the microenvironment will promote the expression of inhibitory receptor ligands by nearby cells to avoid a rush of the immune response [8].

One of the main tools in the arsenal of the immune system is a group of specialized cells known as lymphocytes. Lymphocytes are divided into three main populations, different in terms of function and surface markers: T lymphocytes, B lymphocytes, and “natural killer” (NK) lymphocytes. The specific surface markers for T, B, and NK cells are CD3, CD19, and CD16+56, respectively. CD3 is expressed in 60–80% of normal lymphocytes in peripheral blood and 65–85% in thymocytes. T lymphocytes perform complex functions—both as an effector of the cell-mediated and regulatory immune response—through humoral factors they secrete, called lymphokines. They perform the following functions: regulation of the immune response, control the cells that express non-self-molecules on their surface, and, last but not least, mediation of the reactions of delayed hypersensitivity [9]. B lymphocytes participate in the specific humoral immune response. CD19+ B lymphocytes can be activated and differentiated in mature plasma cells when there are helper T cells and interleukin-1 regulatory factors that generate antigen-specific immunoglobulin. CD19+ B lymphocytes inhibit the activity of helper T lymphocytes, indirectly inhibiting B cell differentiation. It negatively regulates the humoral and cellular immune response of cellular T subsets. “Natural killer” (NK) cells mediate natural cytotoxicity, independent of antibodies, by directly intervening in the elimination of tumor and virally infected cells (for example, by activating phagocytic cells) [7]. CD4+ lymphocytes can proliferate by activating other types of immune cells that produce a direct immune response or provide help to other lymphocytes. The role of cytotoxic T lymphocytes is to destroy infected target cells. Suppressor T lymphocytes are a controversial subpopulation of T lymphocytes. These lymphocytes have inhibitory effects on the immune response [10, 11].

Currently, the anticheckpoints that are used in oncology target inhibitory receptors are CTLA4 (cytotoxic T-lymphocyte-associated antigen 4) and PD-1 (programmed cell death protein 1) and its PD-L1 ligand. CTLA4 and PD-1 are two such checkpoints, which are expressed on activated T cells and attenuate the effector T cell response. The first generation of anticheckpoints targets CTLA4. CTLA4 is expressed in T cells, cytotoxic CD8+, and also at the level of CD4+ auxiliary T and regulatory T (TReg) cells. It intervenes early in T cell activation in the secondary lymphoid organs, during the presentation of the tumor antigen by the naive T lymphocyte dendritic cells, by inhibiting lymphocyte activation in T effectors. CTLA4 molecules are present inside intracellular vesicles and are only transported to the surface of the lymphocyte only when the TCR-specific antigen is recognized. It is an early modulator of lymphocyte activation: if the stimulation via the TCR is stronger, more CTLA4 is produced in large quantities. CTLA4, which is an inhibitory coreceptor, has the same ligands as the CD28 activator coreceptor: CD80 and CD86. As CTLA4 has a stronger affinity for these ligands than CD28, it counteracts the activating effect of the CD28 and results in lymphocyte inhibition. The second generation of anticheckpoints targets the PD-1 coinhibitor receptor or one of its PD-L1 receivers. The way of the programmed cell death protein 1 (PD-1) is another negative feedback that has the peculiarity of acting later in the process of lymphocyte activation at the level of peripheral tissues and the tumor microenvironment. While CTLA4 regulates the early activation of the naive T lymphocyte in the lymph node, the PD-1 receptor acts on the activation of the T lymphocyte during its effector phase on contact with the tumor [12–15].

There has been extensive research on novel biological markers that are specific to the mechanism of action of immune checkpoint inhibitors that may predict response to therapy, and these markers have been recently and extensively reviewed.

## 2. Materials and Methods

In this study, we included 20 patients, all of which were hospitalized and diagnosed with advanced renal cancer, lung cancer and melanoma (stage IV) regardless of the status of programmed death ligand 1 (PD-L1). The patients were all treated with immunotherapy using nivolumab (240 mg) or pembrolizumab (200 mg) alone. Each cycle is of 14 days and starts from the day the patient received nivolumab and of 21 days starting from the day the patient received pembrolizumab. We assessed the state of illness every cycle by checking the blood tests and radiological test after 8–12 weeks from the first day of treatment. All patients were evaluated by imaging according to the criteria of RECIST 1.1 and according to the protocol of nivolumab and pembrolizumab administration. Each patient received at least 3 doses of pembrolizumab and 4–6 doses of nivolumab. The age range was from 40 to 77 years (median, 60.5 years). Informed consent was obtained from all patients, and all procedures performed in the study involving human participants were in accordance with the ethical standards. If patients had brain metastases at study entry, they were initially stabilized by radiation therapy.

Peripheral blood samples (5 mL) were collected from 20 patients in every cycle before treatment with anti-PD-1 antibodies. We collected three blood samples in total from each patient. The second sample was taken 14 or 21 days after the first cycle of PD-1 antibody treatment. The study complied with the Declaration of Helsinki and was approved by the Institutional Review Board Ethics Committee of OncoFort Hospital, Bucharest, Romania, no. 6133/02.09.2018.

### 2.1. Flow Cytometry

We used flow cytometry technique for analyzing the modifications of different subsets of lymphocytes in relation to the response to immunotherapy.

The principle of the method is as follows.

Flow cytometry is a technique that allows the measurement of the properties of suspended biological particles running through a flow system. The term consists of three other basic terms (cyto = cell, metric = measurement, and flow = referring to the flow of suspended particles), and the suspension represents the state in which the particles are dispersed in a fluid. The marked samples are purchased from the flow cytometer. During the acquisition of the sample, the cells move one by one through a laser beam scattering the incident light (a phenomenon called light scattering). At the same time, fluorochrome-labeled cells emit fluorescence generated by fixing labeled reagents. These scattering and fluorescence signals, detected by the instrument, provide information about the cell size (FSC), its internal complexity (SSC), and the relative fluorescence intensity. Three distinct stages of processing the scattered light and fluorescence emitted by the analyzed particles can be described. In the first stage (optical processing), the light coming from the cells is collected by means of optical devices, being then selected by filtering certain wavelengths that are directed to the detectors. In the second stage (optical-electronic conversion), at the level of the detectors, an electrical signal is generated proportional to the intensity of the captured optical signal. In the third stage (electronic signal processing), the electrical impulses produced by the detectors are amplified. The electrical signals are then converted into digital values that can be processed, stored, and displayed by the computer attached to the system. The cells appear graphically as agglomerations of dots forming distinct cell populations (lymphocytes, T lymphocytes, Th lymphocytes, T/C lymphocytes), and the device records the values in the form of percentages of sets and subsets of the tested cell populations [16].

### 2.2. Statistical Analysis

The data are shown as median values, with error bars representing the range from the minimum value (lower bar) to the maximum value (upper bar). The results were analyzed using the Kruskal–Wallis nonparametric test (ANOVA). Statistical analysis and graphing were performed using GraphPad Prism, version 5.0 (GraphPad Software, Inc.). Results were considered statistically significant at *P* < 0.05.

## 3. Results

### 3.1. Characteristics of Patients

In this study, we included 20 patients diagnosed with advanced malignant renal, lung, and melanoma tumors (stage IV) with a mean age of 60 (extreme: 40–77 years). Gender distribution was equal, that is, 10 female patients and 10 male patients were included (Table 1).

Of the total number of patients, there were 2 women and 6 men with lung cancer group, 8 women and 2 men with melanoma, and only two male patients with kidney cancer (Table 2).

The median age of the patients diagnosed with lung cancer was 64 years; for melanoma, it was 59 years for women and 55 years for males; and it was 55 years for kidney cancer. Based on the clinical evaluation, 12 patients had stable disease, 6 patients had progressive disease, and 2 dead.

We aimed to quantify the extent of lymphodepletion or increase of T lymphocytes following immunotherapy. We found a mean decrease of CD4/CD8 ratio and low values of T lymphocytes in two patients before and after the first administration of immunotherapy, which was associated with a poor prognosis.

The two patients included in the study who died after a first administration of immunotherapy presented the following values of T lymphocytes: 19.14% CD3+, 8.11% CD4+, and 9.30% CD8+.

Interesting is the case of the second patient, who died after 2 immunotherapy administrations and who presented instead the low CD4/CD8 ratio of 0.45 before the first administration and 0.43 after the second administration.

We dynamically tested the expression of immune biomarkers by flow cytometry before the first administration and after the other administrations of immunotherapy for all patients.

We analyzed the variation in trends of each biomarker. Some of the biomarkers on circulating T cells showed a difference between groups at the before treatment time point, and we believe the reason for this is that these patients were diagnosed with different types of cancer and had previously received different chemotherapeutic agents in lung and renal cancer and target therapy for BRAF mutation in melanoma, which influenced the initial state of their immune systems. No variants of dynamic evaluations were observed depending on the tumor type or gender.

Immune checkpoint inhibitors have not been associated with severe immune-related adverse events (ir AEs) such as rashes, diarrhea, colitis, hypophysis disorder, hepatotoxicity, and hypothyroidism.

**Figure 1:**
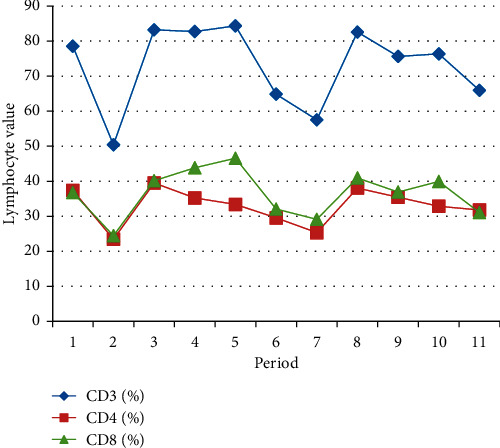
Immune biomarkers evaluated in dynamic patient 1.

**Figure 2:**
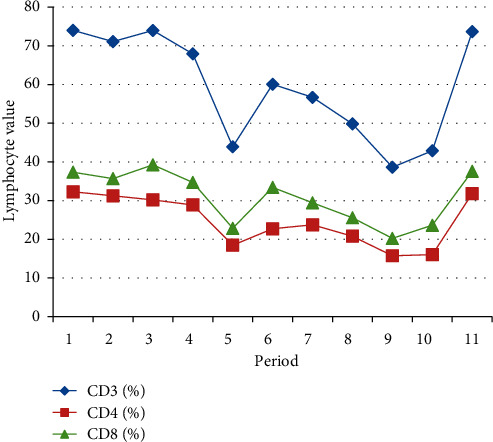
Expression of biomarkers on CD8+ T cells in patient 2.

From the total number of patients, we extracted 2 patients who performed a number of 11 immunotherapy administrations so that we could show the dynamics of T lymphocytes during the treatment, which is exemplified in Tables 3 and 4 and Figures 1 and 2.

A 77-year-old female patient with metastatic melanoma, without BRAF mutation and previous treatment, is the first case presented and treated with nivolumab (240 mg) as a first line at an interval of 14 days.

The second patient is a 58-year-old male diagnosed with advanced lung cancer. Nivolumab was given as a second-line treatment, and cytotoxic chemotherapy was given as the first-line treatment.

We subsequently subtracted the individual expression values from the mean of the appropriate time point and drew a trend curve to assess changes in expression, which can be seen in the figures above.

In graphical representation, it is observed that the CD3+, CD4+, and CD8+ counts followed the same trend initially downward then upward.

We evaluated the absolute numbers of the above-mentioned immune cell subsets to confirm their impact on OS and response to treatment. In agreement with the data shown in Figures 1 and 2, patients characterized by a high number of circulating CD3+ lymphocytes displayed a significantly longer OS.

**Figure 3:**
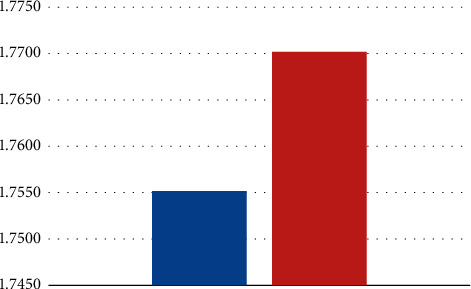
Changes in the CD4/CD8 ratio before and after immunotherapy.

The change in the CD4/CD8 ratio before and after one course of immunotherapy is shown in Figure 3. Among the total cohort of 20 patients, there was no significant difference in the CD4/CD8 ratio, only two who had lower ratios before and after the first course of immunotherapy. The analysis of the number of patients with a decrease or increase in CD4 and CD8 T cell numbers after one course of immunotherapy revealed that a significant increase in CD8 T cell number with a change in CD4 T cell number was seen in 18 patients after the first course of immunotherapy.

These results (Figure 3) reveal that the tendency of the CD4/CD8 ratio was to increase. The tendency of CD4/CD8 ratio to decrease depended mostly on an increase in CD8 T cells and not on CD4 T cell numbe. As CD8 T cells are cytotoxic and kill cancer cells, a significant increase in the number of CD8 T lymphocytes may improve the anticancer suppressive activity of the patient's immune system during immunotherapy.

## 4. Discussion

Immune checkpoints are essential for the maintenance of immune self-tolerance. Tumor cells can escape from these immune-regulatory mechanisms, allowing cancer to continue to grow and metastasize.

In this research, we have focused on 3 types of cancer—renal cancer, lung cancer, and melanoma—with impact on the population. In this study, we obtained a blood-based immune signature on circulating T cells before and early during immunotherapy. Lung cancer is the most commonly occurring cancer in men and the third most commonly occurring cancer in women. There were 2 million new cases in 2018 [5]. Melanoma is the deadliest of skin cancers and is one of the most common skin cancers found among young adults [7]. Recent insights into the molecular and cellular mechanisms underlying cancer development have shown that immune cells functionally regulate cancer development and progression. One of the main tools in the immune system arsenal is a group of specialized cells known as lymphocytes [8].

The evaluation of the immune system is difficult to perform due to the multitude of factors involved in patients diagnosed with cancer [17]. At present, the immune response is long studied because of the fact that we are in the era of immunotherapy [18–20]. The immune response depends on the genetics of each individual and the previous therapies used without success [21, 22]. Previous studies have assessed the expression of immune biomarkers; however, the reproducibility of these tests remains to be determined [23–25].

The prognostic importance of the expression of PD-1 on tumor-infiltrating lymphocytes and circulating T cells is demonstrated in a number of studies [26–28]. Zheng showed that high PD-1 expression on peripheral CD4+ T cells is associated with inferior clinical response in non-small-cell lung carcinoma patients who received anti-PD-L1 treatment [29]. Two further studies found that, compared with healthy individuals, cancer patients had high levels of PD-1 expression [30–33].

Our study is the first to describe the variation tendency of biomarker expression during the cycles of immunotherapy in different localizations of the primary tumor, and we constructed a simple model to predict the therapeutic effect of immunotherapy.

By analyzing the cells of the immune system, we will be able to determine if the patient responds to the treatment or not. Another novelty of this study is that we are in the peak period of immunotherapy and no markers have been found to track the response to treatment, the doctor stops treatment only on the basis of imaging investigations, and the evaluation of the cells of the immune system is useful.

Evaluation of T lymphocytes in the blood and their correlation with the percentage of T lymphocytes in the tumor lymphocyte infiltrate will show us how the immune system responds to the disease and what we can develop to get a better response.

Previous studies have shown that the ratio of CD4/CD8 T cells reflects the status of the immune system and can independently predict mortality from all causes. Shah et al. [32], in his study, reported that the low CD4/CD8 ratio was significantly associated with the worse prognosis of patients with cervical carcinoma, and in the study of Chang-Juan Tao, from 2016, it was shown that the value of the higher CD4/CD8 ratio (≥1.77) was associated with the disease-free interval [33].

The present study showed a statistically significant association between the change in the CD4/CD8 ratio before and after one course of immunotherapy and the downward tendency of the CD4/CD8 ratio during one course of immunotherapy, suggesting an active immunological response against cancer invasion.

There are conflicting literature data on the decrease in the proportion of CD4+T lymphocytes in the blood as a result of tumor invasion, causing CD4+ cells to migrate from the blood to the tumor site. There are studies that claim that tumor eradication is associated with a low number of CD4+ T lymphocytes and studies that claim that an increased percentage of CD4+ T lymphocytes are associated with the response to treatment and eradication of the tumor [33, 34].

There is also evidence that CD8+ T lymphocytes represent the class of lymphocytes that correlate best with favorable overall clinical outcomes, usually infiltrating breast lesions to the greatest extent [34]. It has been widely documented that the presence of high rates of CD8+ T cell infiltration is associated with higher overall survival rates, and we also assessed whether patients with high CD4/CD8 ratios show better OS and/or clinical benefit.

In a previous study involving 678 patients treated with autologous activated lymphocyte immunotherapy, the CD4+ T cell numbers were maintained at almost the same level compared with the levels before immunotherapy, whereas the CD8 T cell number increased significantly the result obtained in our study [35].

There are several different kinds of immunotherapy using different phenotypic proportions of CD4 and CD8 T cells and differing CD4/CD8 ratios. A study which involved 100 patients with metastatic breast cancer who had received direct subcutaneous injection of low-dose IL-2 and administration of oral retinoic acid to prevent tumoricidal activity and were followed up for 3 years showed that the lymphocyte count, NK cell numbers, and the CD4/CD8 ratio were found to increase significantly [36].

One limitation in this study is the impossibility to evaluate PD-L1 expression for patients with melanoma and kidney cancer.

It would be interesting to correlate the imaging data evaluation of peritumoral TILS with the values of the T lymphocytes in the blood.

## 5. Conclusion

In general, the decrease in the immunoreactive threshold in the neoplastic patient affects the numerical balance of lymphoid cells in the peripheral circulation, deviating from the normal percentage values of different cell sets and subsets, especially total T lymphocytes (CD3+), “helper” T lymphocytes (helper CD4+), and cytolytic and “suppressor” T lymphocytes (CD8+). Therefore, the determination of the proportions of CD3+, CD4+, and CD8+ cells is useful in the evaluation of monitoring patients with immune status affected by cancer. The values of the obtained parameters can be correlated either with the immunosuppressive state given by the tumor, its stage of expansion, and consistency of the immunoreactive means of the body, or with the immune status resulting from the application of immunosuppressive treatments (chemo-, radio-, or immunotherapy).

The evaluation of the cells of the immune system in relation to the tumor and immunotherapy may lead to a better understanding of the pathogenic mechanisms and the identification of prognostic and predictive factors that will more effectively model the therapeutic approach.

## Figures and Tables

**Table 1:** Distribution of tumor location by sex.

Count of age	Column labels		
Row labels	Female	Male	Grand total
Lung	2	6	8
Melanoma	8	2	10
Renal		2	2
Grand total	10	10	20

**Table 2:** Distribution of tumor location by age and sex.

Average of age	Column labels		
Row labels	Female	Male	Grand total
Lung	64.50	64.50	64.50
Melanoma	59.63	55.00	58.70
Renal		55.50	55.50
Grand total	**60.6**	**60.8**	**60.7**

**Table 3:** Evaluation of lymphocyte T values in patient dynamics no. 1.

CD3 (%)	CD4 (%)	CD8 (%)
78.56	37.4	36.79
50.40	23.46	24.46
83.22	39.52	40.15
82.74	35.23	43.85
84.35	33.40	46.58
64.85	29.55	32.07
57.53	25.35	29.13
82.58	38.07	40.95
75.63	35.45	36.93
76.36	32.87	39.95
65.96	31.81	31.10

**Table 4:** Evaluation values in patient dynamics no. 2.

CD3 (%)	CD4 (%)	CD8 (%)
73.99	32.27	37.33
71.08	31.23	35.68
73.96	30.17	39.24
67.92	28.88	34.69
43.92	18.46	22.85
60.06	22.72	33.41
56.67	23.74	29.44
49.82	20.79	25.56
38.61	15.74	20.24
42.88	16.04	23.63
73.67	31.79	37.59

## Data Availability

The data sets used and/or analyzed during the current study are available from the corresponding author on reasonable request.
